# Multiple Hemolymphangioma of the Visceral Organs: A Case Report and Review of the Literature

**DOI:** 10.1097/MD.0000000000001126

**Published:** 2015-07-13

**Authors:** Deng-Yong Zhang, Zheng Lu, Xiang Ma, Qiu-Yue Wang, Wang-Liang Sun, Wei Wu, Pei-Yuan Cui

**Affiliations:** From the Department of Hepatobiliary Surgery, First Affiliated Hospital of Bengbu Medical College (D-YZ, ZL, XM, W-LS,WW, P-YC); Department of Bengbu Medical College, Bengbu, Anhui, China (Q-YW).

## Abstract

Hemolymphangioma is a rare disease with malformation of both lymphatic and vascular vessels. Few cases of hemolymphangioma occurring in the rectum, small intestine, pancreas, esophagus, and other organs have been reported. Nevertheless, multiple hemolymphangioma of the visceral organs are extremely rare. We report a 25-year-old female with a significantly enlarged spleen full of multiple-rounded lesions. Curiously, the splenic flexure and even retroperitoneum had many lesions. The patient recovered well after splenectomy and the pathologic diagnosis of spleen was hemolymphangioma with abnormal lymphatic and blood vessels with polycystic spaces.

Usually, it is hard to cure this disease. We should take much more consideration into the diagnosis, treatment, and even pathogenesis, even though it is a benign lesion.

## INTRODUCTION

Hemolymphangioma is a rare benign primary neoplasm and is usually a space-occupying lesion of the parenchyma of organs. So far only a few cases occurring in the small intestine, spleen, rectum, chest wall, mediastinum, adrenal gland, pancreas, duodenal, extremities, or orbit have been reported.^1–10^ Hemolymphangioma often occurs in young person and newborns and infants. Its tissue often originates from congenital malformations of the vascular and lymphatic system. Until now, multiple hemolymphangioma of the visceral organs have rarely been reported. In this article, we describe a 25-year-old female with massive splenomegaly caused by diffuse hemolymphangioma and finally diagnosed as multiple coeliac hemolymphangioma by imageological examinations.

## CASE PRESENTATION

### Patient Information

The 25-year-old female was admitted to our hospital in October 2014 for progressive splenomegaly during the past year. Multiple spleen cysts had been detected by ultrasound examination of the abdomen 6 years ago; however, no treatment was taken after that (Original ultrasound report was missing, so it is hard to judge whether other organs had space-occupying lesions). She had no abdominal pain, fever, hemoptysis, hematemesis, or other gastrointestinal bleeding symptoms. There was no history of trauma, weight loss, nor family history of cancer.

### Clinical Findings

**FIGURE 1:**
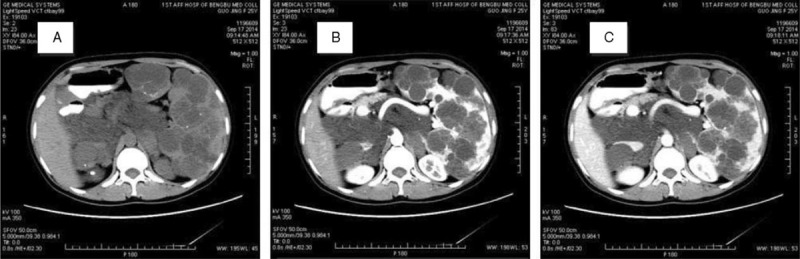
Computed tomography scan demonstrates multiple cystic masses of the abdomen. (A) Plain, (B) arterial phase, (C) delayed phase.

**FIGURE 2:**
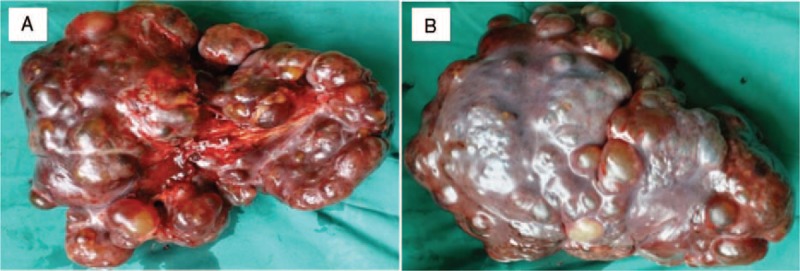
Splenic parenchyma containing multiple cystic masses. (A) The obverse, (B) the reverse.

**FIGURE 3:**
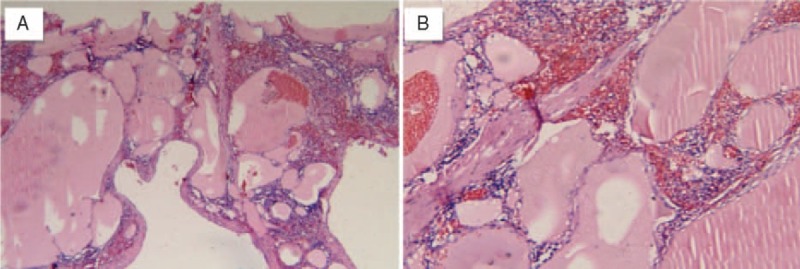
Pathological examination of the splenic hemolymphangioma (hematoxylin and eosin staining). (A) Tortuous dilated lumen blood vessels communicating with each other, vessel lumen filled with erythrocytes and weakly staining fluid, consisting of loose connective tissue and some lymphocytes (100×). (B) Under the high magnification, vessel lumen inner lining flat endothelial cell, full of blood, separated by collagenous fiber mesenchyme.

Physical examination showed a soft left upper-quadrant abdominal mass with mild tenderness. Recently, she found an abdominal mass and subsequent ultrasound examination of the abdomen revealed a significantly enlarged spleen (thickness 10 cm, length 26 cm). Multiple rounded lesions up to 5.8 cm in diameter were seen throughout the spleen. The right abdomen intestinal canal and mesangium had multiple anechoic regions up to 2.1 cm in diameter, some of which integrated into the network. Abdominal computed tomography (CT) was then performed. CT scan showed multiple heterogeneity abnormal density shadow in different size distributed in porta hepatis, spleen, and retroperitoneal clearance invading neighboring liver and the cyst wall with mild enhancement after being contrast enhanced. There were multiple gelatin lesions with unobvious enhancement throughout the retroperitoneum, compressing other abdominal organs (Figure 1). The spleen measured 27 cm in the craniocaudal diameter and 11 cm in the sagittal plane. The CT scan diagnosis was splenomegaly, celiac pseudomyxoma, with liver and spleen invasion paramount for consideration. Routine blood examination was unremarkable except with a platelets count of 90 × 10^9/L. Tumor markers like CA19-9 (shine) 45.37 IU/mL and CA125 (shine) 54.40 IU/mL were a little higher than normal upper range, with others like CEA, AFP, CA153 normal. These findings were thought to represent multiple space-occupying lesion in enterocoely and for the origin of the lesions, spleen, retroperitoneum, or other visceral organ should be fully taken into consideration. Under these circumstances, lacking of biopsy, it was unknown if the benign or malignant and so the patient's symptoms and the tumor location were taken into consideration, resulting in planning an exploratory laparotomy (preparation splenectomy). In operation, we found the spleen was 28 cm × 24 cm × 15 cm in size (Figure 2), with multiple cystic lesions. The liver (left lobe closed to spleen) and porta hepatis had no invasion lesions. After removing the spleen, we saw the retroperitoneal tumor extending to the left colon and small bowel mesenteric. Because the right retroperitoneum was full of cystic lesions, we could not resect completely, so a splenectomy was performed. The surgery took 2 hours with an intraoperative blood loss of 100 mL. Macroscopically, the spleen showed a soft tissue mass and its parenchyma consisted of lymphatic and blood vessels with multiple cystic spaces. The pathological diagnosis was multiple splenic hemolymphangioma (Figure 3). Microscopically, hemolymphangioma was composed of abnormal lymphatic and blood vessels, with lumen size, thin wall, composed of fibrous tissue, and dyed red lymph and blood cells within the lumen. The patient recovered unevenly and was discharged on postoperative day 5.

## DISCUSSION

### Description of Hemolymphangioma

The etiology of hemolymphangioma is always considered to be congenital malformation of the vascular system and mesenchymal tissue.^11^ The abnormal venolymphatic communication between the dysembryoplastic vascular tissue and blood circulation could be the reason for the formation of this tumor, as well as the obstruction of lymph drainage or lymphatic damage.^7^ Most studies consider hemolymphangioma as a benign tumor due to its good prognosis, but some studies found that it can invade the adjacent structures,^12–13^ the same as our case. Diffuse or giant celiac hemolymphangioma is very rare and even rarer is the involvement of the spleen. There have been only 2 cases reported on PubMed, 1 was a giant celiac hemolymphangioma,^12^ the other is a multiple celiac hemolymphangioma.^14^ And characteristics were shown in Table 1.

**TABLE 1:**
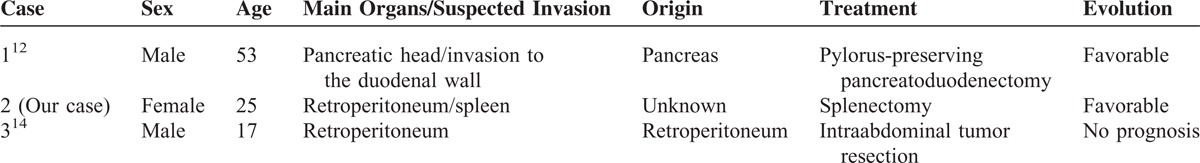
The Characteristics of 3 Patients With Multiple Hemolymphangiomas of the Visceral Organs

### Splenic Tumor

Splenic primary benign tumors are extremely rare and account for less than 0.007% of all tumors identified pathologically and surgery.^15^ Primary splenic tumors were classified into 4 types by Morgenstern et al^16^: tumor-like lesions, vascular tumors, lymphatic tumors, and nonlymphatic tumors. The splenic vascular tumors include hemangioma, lymphangioma, hamartoma, hemolymphangioma, and so on. Splenic hemangioma is the most common benign tumor and the most uncommon is hemolymphangioma. There were only 3 cases reported on spleen previously (PubMed),^2,17,18^ and the characteristics are shown in Table 2. Histologically, hemangioma distributed throughout the organ parenchyma and filled with endothelial cells expressing vimentin, factor VIII, and CD 31, but not CD8. Lymphangioma is a congenital malformation of the lymphatic system, predominantly occurring in children. According to the congenital dilated lymphatic channels, lymphangioma can be classified into 3 subtypes: capillary (super-microcystic), cavernous (microcystic), and cystic (macrocystic).^19^ The clinical presentation of splenic benign tumors is variable. Children tend to show shorter durations and acute symptoms, but adult tend to have mild and nonspecific symptoms, like pain or discomfort in the left upper quadrant, abdominal distension, and a palpable mass.

**TABLE 2:**
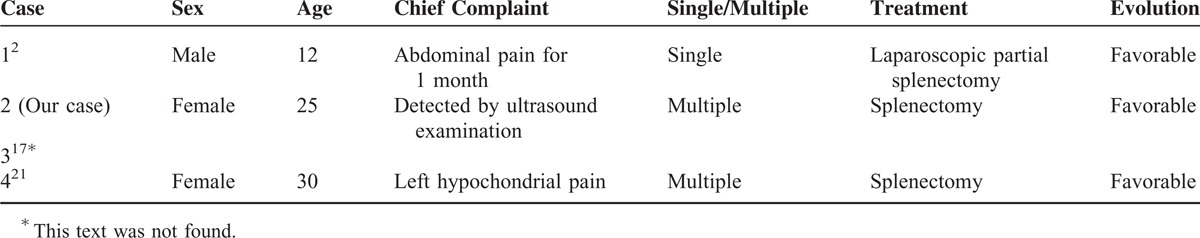
The Characteristics of 4 Patients With Splenic Hemolymphangioma

### Clinical Findings and Diagnosis of Splenic Tumors

The treatment of splenic tumors should depend on the size and location of the tumor, the patients’ symptoms and signs, and the methods including complete surgical resection, drainage, and irradiation. However, surgical resection should be the treatment of choice when the diagnosis is established as a growing number of complications would occur overtime, such as infection, tumor rupture hemorrhage, and anemia, and resulting large tumor growth may prevent marginal negative surgical removal completely.^20^ Many surgeons consider splenectomy as the treatment of choice as it is a feasible, reproducible, and safe way to cure benign splenic tumors. However, for the multiple splenic lesions or tumors with unknown pathology before surgery, exploratory laparotomy is required. Patient can live for a long time with no symptoms but when the tumor has grown enough, it can cause serious symptoms. The diagnosis of this tumor is very difficult because of its rarity and the absence of clinical symptoms. A palpable abdominal mass and left upper quadrant discomfort are the most common symptoms of this tumor. There are some uncommon symptoms such as vomiting and satiety which are caused by the tumor in the later period. Laboratory tests like routine blood examinations and tumor markers (such as CA199, CEA, AFP, PSA, and CA125) are frequently within the normal range. Ultrasonography is recommended for screening abdomen mass early and CT and magnetic resonance imaging (MRI) are useful to measure the extent of the tumor and in estimating the relationship with the important blood vessels in order to design the best surgical plan. The CT scan of splenic hemolymphangioma always shows many cysts with heterogeneous enhancement (Figure 1). Typically a splenic tumor image has a multiple diffused circular type with low density and, based on the composition of blood vessels and lymphatics, the edge of lesions makes a different image with many more vessels making an unclear, uneven enhancement image; more lymphatics make a low density, clear boundary on a CT scan.^10^ In our case, it was initially diagnosed as a lymphangioma based on CT findings. Three-dimensional reconstruction has been proven safe and may result in decreasing the risk of bleeding and increasing safety during surgery, but it could not be completed in our case because the blood vessels were oppressed by tumors.

Biopsy of visceral organs has a high risk of massive bleeding, damage to the surrounding organs, or cancer migration, so it is not the preferred way for defining the histological type of the spleen tumor. It is especially more difficult for multiple space-occupying lesions of intraabdominal involvement of the spleen. In the study by Zhang et al's,^4^ a biopsy for a chest wall hemolymphangioma under the guidance of CT was taken without any complications. Even so, the author commented that fine-needle aspiration biopsy may lead to many bad consequences, especially inducing tumor metastasis. It is necessary for radiologists to establish accurate diagnosis criteria to recognize these lesions to guide clinical treatment. Clinical differential diagnoses include splenic abscess, cyst, lymphangioma, hemangioma, fibrosarcoma, and metastatic tumors. The clinical diagnosis is based on a combination of symptoms and radiological, and the final diagnosis depends on histopathological findings.

### Treatments for Splenic Tumors

Splenectomy is the treatment of choice for solitary splenic tumors, but the optimal treatment strategy of splenic tumor remains controversial. The surgical methods are based on the size of tumor, its relationship to the main vessels and the remaining amount of healthy splenic tissue. Some people prefer laparoscopic partial splenectomy as being good for patients especially for children with hematological diseases or focal splenic tumors.^21^ This method preserves the spleen's immune role. Most benign tumors are easy to resect due to its small size and slightly adhesion with the surrounding organs. For malignant tumors, splenectomy and regional lymphadenectomy or even combined multiple organ resection are needed. In our case, the retroperitoneum was full of cystic lesions with the same characters as spleen. Considering the anatomical location of lesions, surgical difficulty, and postoperative complications, we did not remove the retroperitoneal cyst. For patients with multiple celiac space-occupying lesions, sufficient preoperative assessment is necessary. On one hand, you should take care of the origin of lesions and on the other hand, imaging examinations like CT, MRI, 3-dimensional reconstruction, and even the biopsy can be helpful in the evaluation and to avoid accidents in surgery. Some studies prefer MRI as the important means for diagnose as well as the immunohistochemical identification of factor XIII.^14^ If the origin of lesions is clear, the surgeon should excise both the main and the invaded lesion organs.^12^ In addition, the basic condition of the patient, like the age, underlying diseases, and others, is commonly evaluated before surgery. It is difficult for multiple intraabdominal space-occupying lesions to be resected completely, and so it is reasonable to just remove the main organs and some operable invading lesions. Incomplete excision is the reason for postoperative recurrence, as well as an unresolved subject for surgeons clinically. In our case of multiple intraabdominal hemolymphangioma, splenectomy was done to remove the main diseased organ, the retroperitoneal occupying lesions could not excised completely due to its anatomical position. Although this patient left hospital in 5 days after surgery and live without tumor recurrence and operative complications up to date (about 2 month), regular follow-up is highly recommended.

### Uncited references

^20^^.^
